# Continuing Professional Development—Radiation Therapy

**DOI:** 10.1002/jmrs.70108

**Published:** 2026-07-05

Maximise your continuing professional development (CPD) by reading the selected article and answering the five questions. Please remember to self‐claim your CPD and retain your supporting evidence. Answers will be available via the QR code and published in JMRS – Volume 73, Issue 4, December 2026.

## Comparative Evaluation of Four Stereotactic Radiosurgery Planning Approaches for Treatment of Posterior Choroidal Melanoma

Robert Nigro, Kenton Thompson, Cathy Markham, Adam U. Yeo, Daniel Sapkaroski, Claire Phillips, https://doi.org/10.1002/jmrs.70076.

1. Which treatment planning approach demonstrated the lowest median gradient index in this study?
  - Dynamic conformal arc therapy (DCAT)
  - HyperArc
  - Brainlab Elements (BE)
  - Gamma Knife
2. Why is retrobulbar anaesthesia required for fixed‐frame single‐fraction Gamma Knife treatment in some centres?
  - To immobilise the eye completely.
  - To enhance MRI contrast.
  - To reduce dose to the PTV.
  - To reduce high‐dose gradients.
3. In this planning study, why did the treating radiation oncologist aim to keep the 20% isodose (10 Gy) within the bony orbit while maintaining organ‐at‐risk (OAR) constraints?
  - To reduce the dose to the eyelid and lacrimal gland.
  - To minimise low‐dose irradiation of normal brain tissue.
  - To increase the PTV margin and improve target coverage.
  - To account for eye movement during treatment.
4. A treatment plan achieves a Paddick conformity index (PCI) of 0.76 using BE and 0.65 using DCAT for the same target volume. Based on the findings of this study, how should these results be interpreted?
  - DCAT provides superior OAR sparing due to the lower PCI.
  - PCI does not influence dose distribution outside the target.
  - Improved conformity with BE is associated with poorer OAR sparing.
  - BE provides superior conformity, which is associated with improved brain dose sparing.
5. Gamma Knife demonstrated favourable dosimetric results in this study. Which factor most strongly limited its suitability for clinical implementation?
  - Difficulty achieving adequate target coverage.
  - Prolonged treatment times compared with linac‐based techniques.
  - Lack of a reproducible method for fractionated eye immobilisation and monitoring.
  - Excessive dose to the contralateral eye.

## Answers


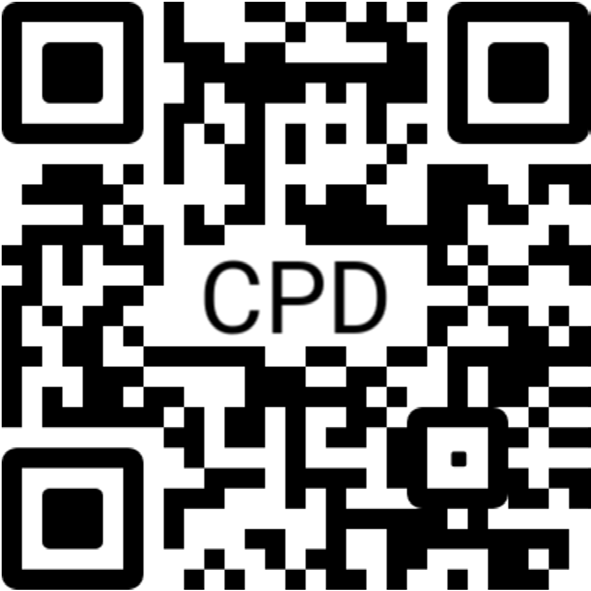
Scan this QR code or https://www.surveymonkey.com/r/JMRS_Sept2026_RT to find the answers.
